# Phylogeographical Analyses of a Relict Fern of Palaeotropical Flora (*Vandenboschia speciosa*): Distribution and Diversity Model in Relation to the Geological and Climate Events of the Late Miocene and Early Pliocene

**DOI:** 10.3390/plants11070839

**Published:** 2022-03-22

**Authors:** Samira Ben-Menni Schuler, Hammadi Hamza, Gabriel Blanca, Ana Teresa Romero-García, Víctor N. Suárez-Santiago

**Affiliations:** 1 Department of Botany, Faculty of Sciences, University of Granada, 18071 Granada, Spain; samira@ugr.es (S.B.-M.S.); gblanca@ugr.es (G.B.); atromero@ugr.es (A.T.R.-G.); 2 Arid and Oases Cropping Laboratory, Arid Area Institute, Medenine 4119, Tunisia; hamzapalmier@yahoo.fr

**Keywords:** fern phylogeography, *gapCp* gene, palaeotropical flora, plastid DNA, refugia, relict fern, species distribution modelling, Tertiary, *Vandenboschia speciosa*

## Abstract

Fern phylogeographic studies have mostly focused on the influence of the Pleistocene climate on fern distributions and the prevalence of long-distance dispersal. The effect of pre-Pleistocene events on the distributions of fern species is largely unexplored. Here, we elucidate a hypothetical scenario for the evolutionary history of *Vandenboschia speciosa*, hypothesised to be of Tertiary palaeotropical flora with a peculiar perennial gametophyte. We sequenced 40 populations across the species range in one plastid region and two variants of the nuclear *gapCp* gene and conducted time-calibrated phylogenetic, phylogeographical, and species distribution modelling analyses. *Vandenboschia speciosa* is an allopolyploid and had a Tertiary origin. Late Miocene aridification possibly caused the long persistence in independent refugia on the Eurosiberian Atlantic and Mediterranean coasts, with the independent evolution of gene pools resulting in two evolutionary units. The Cantabrian Cornice, a major refugium, could also be a secondary contact zone during Quaternary glacial cycles. Central European populations resulted from multiple post-glacial, long-distance dispersals. *Vandenboschia speciosa* reached Macaronesia during the Pliocene–Pleistocene, with a phylogeographical link between the Canary Islands, Madeira, and southern Iberia, and between the Azores and northwestern Europe. Our results support the idea that the geological and climate events of the Late Miocene/Early Pliocene shifted Tertiary fern distribution patterns in Europe.

## 1. Introduction

Palaeotropical flora [1], predominantly evergreen and thermophilous plants, populated the Northern Hemisphere, occupying a mid-latitude climate belt on both coasts of the Tethys Sea from the Late Cretaceous to the Late Miocene [2,3]. In Europe, a lauroid-type flora originated and developed with the herbaceous layer composed mainly of ferns [4,5,6,7,8]. The gradual (sub)tropical climate deterioration during Tertiary, especially from the mid-Miocene onwards, together with Pleistocene glaciations, caused the decline of this flora, which contracted to the south and west of Europe, where the climate remained suitable (e.g., [6,9,10,11,12]). Moreover, during the Pleistocene glacial maxima, many temperate species not only found refugia at lower latitudes but also persisted in refugia at higher latitudes close to, or even within, the limits of the ice sheets [13,14]. From all these refugia, temperate and palaeotropical species expanded their populations, even recolonizing Europe, during post-glacial periods [13,15,16].

The dynamics of population contraction and/or expansion, forced by historical geological and climatic events, have influenced the distribution and diversity patterns of the species, leaving a genetic trace in their populations which can be identified by phylogeographical analyses [14]. In plant phylogeography, most studies examine the effects of Pleistocene glaciations on the plant distributions, while few studies address the history of ancient taxa, such as that of the lauroid forest, or analyse the processes that shaped their distribution patterns (e.g., [17,18,19,20,21,22,23]). Few phylogeographical studies on ferns are available (e.g., [24,25,26,27,28,29,30]) and, to date, only one phylogeographical study, including a time-calibrated phylogeny, has addressed the biogeographical history of a presumed Tertiary fern species [30]. Many European ferns are considered Tertiary relicts, localized in shelters having similar microclimatic conditions to those of that time, as those found along the European Atlantic Coast and Macaronesia [5,31,32,33,34,35]. The distribution pattern of these ferns, the antiquity of their lineages, and the biological peculiarities that determine their colonization dynamics, make them suitable species to explore for insights into the responses of European plants to past climate change and the genetic fingerprint left by these changes. 

*Vandenboschia speciosa* (Willd.) G. Kunkel (the Killarney fern; Hymenophyllaceae) is a Macaronesian–European endemic fern, which is thought to have belonged to the Tertiary laurel forests [7]. One of the most remarkable features of this species is that both phases of the life cycle (sporophyte and gametophyte) are perennial and can propagate vegetatively (making it a unique fern in Europe [36]). Therefore, mixed and independent gametophyte populations occur throughout its distribution [37]. Moreover, gametophytes differ from sporophytes in ecological tolerances withstanding drier and darker conditions (such as those within caves and caverns [38,39]), under which they can develop and reproduce in a vegetative way without the need of a sporophyte [40]. Both the sporophyte and gametophyte appear disjunctly in the European Atlantic coast and the Macaronesian islands (Azores, Canaries, and Madeira). In these areas, both generations could develop a normal fern life cycle [41]. As the species entered the continent or expanded in latitude (to Northern and Eastern Europe, reaching Scotland and Poland, respectively), the sporophyte generation disappeared and only the gametophyte remained.

Thus far, only an approximation has been made to the phylogeographical study of *V. speciosa*, using PCR-RFLPs [42], and a few studies geographically limited on genetic diversity using allozymes [37,40,41] and microsatellites [29]. Briefly, Rumsey et al. [42] reported the presence of two chloroplast haplotypes (differing in only one change)—one being a haplotype distributed in the south of the distribution range (Iberian Peninsula, Italy, Madeira, and the Canary Islands) and the other one in the north (Azores, Central and Northern Europe)—and the existence of mixed populations in the European Atlantic coast (Britain, Ireland, and NW France). The authors suggested that this result might reflect the retreat and expansion of *V. speciosa* populations during the Pleistocene ice ages. Rumsey et al. [37,40], using allozyme multilocus phenotypes distribution, analysed the genetic differentiation between/within populations of Central Europe (the Vosges and Germany) and Scotland (UK); Rumsey et al. [41] used this information, together new data for three Andalusian and two Italian populations, to try to identify which areas have acted as a refuge and which ones can be interpreted as resulting from recent colonization. The low diversity observed, practically null at the intrapopulation level, in the Scottish and Central European populations led the authors to propose the post-glacial recolonization of these areas; while, due to their greater diversity, the Andalusian and Italian localities were considered refuge areas [41]. Ben-Menni Schuler et al. [29] analysed, with microsatellites, the genetic diversity of the Andalusian sporophyte populations, and found a moderate genetic diversity at the regional scale but low diversity at the population level. These authors suggested that the ancestral gene flow and genetic drift have played a key role in shaping the genetic structure of Andalusian *V. speciosa* populations. Despite these studies, the origin and evolutionary history of *V. speciosa* remains elusive. In the present study, we used plastid DNA (ptDNA; intergenic spacer *trn*H-*psb*A), low-copy nuclear gene (*gapCp* gene) sequences, and species distribution modelling (SDM) to explore patterns of diversity and distribution of *V. speciosa* throughout Europe and Macaronesia. Within our overall aim, the specific objectives were the following: (1) to test the Tertiary relict hypothesis; (2) to compare the genetic structure between the two life cycle phases; (3) to infer the phylogeographical patterns throughout its whole range and explore the putative impacts of past climate changes in modelling those patterns; and (4) to determine the impact of future climate warming on the species distribution as a whole, considering a possible differential response of gametophyte and sporophyte.

## 2. Materials and Methods

### 2.1. Plant Material

**Figure 1:**
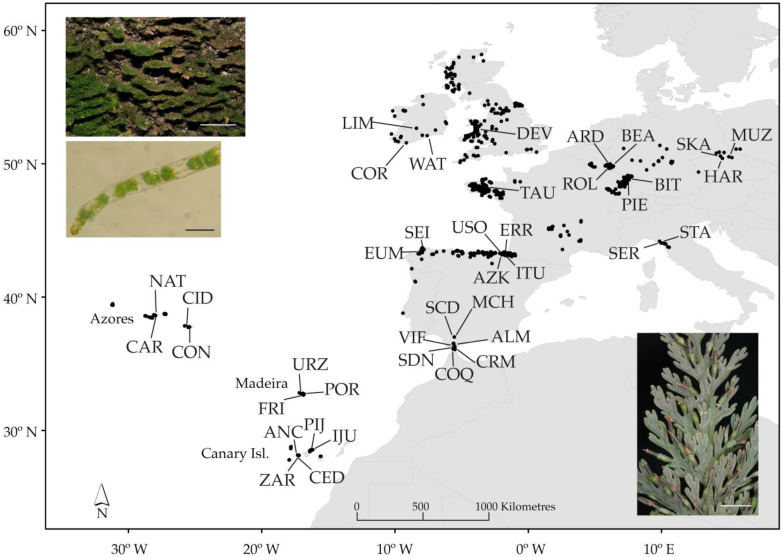
Geographical distribution of *Vandenboschia speciosa*, showing the 40 populations sampled for phylogeographical analyses (see Table S1 for population code) and the location of presence records (black dots) used for species distribution modelling (SDM). Two photographs of the gametophyte, showing the habit (scale bar 5 cm) and a microscopic detail (scale bar 100 μm), are shown in the upper left corner; and in the lower right corner, the detail of a frond with sori is shown (scale bar 5 mm).

Samples of *Vandenboschia speciosa* were taken from 40 populations in 11 geographical regions across its distribution range (Figure 1; Table S1). Twelve populations were formed only by gametophytes, four by only sporophytes (no gametophytes were observed after exhaustive searching), and the remaining were mixed populations. Both gametophyte and sporophyte generations were sampled, including between 1 and 11 individuals per generation and population. A total of 309 individuals were sampled, i.e., 168 gametophytes and 141 sporophytes. Fronds of two *V. boschiana* fresh individuals (provided by F. Rumsey), two *V. davallioides*, and two *V. birmanica* accessions were also sampled, from herbaria, as outgroup species (Table S1).

### 2.2. DNA Extraction, PCR Amplification, and Sequencing

Total genomic DNA was extracted from cleaned filaments of gametophytes, and from fresh, silica dried, or herbaria fronds of sporophytes using the NucleoSpin Plant II kit (Macherey-Nagel GmbH and Co. KG, Düren, Germany).

Plastid DNA for the intergenic spacer *trn*H-*psb*A was amplified by polymerase chain reaction (PCR) in all 309 individuals of *V. speciosa* and three outgroup species; we could not amplify this region in one of the two *V. birmanica* accessions. A previous survey using primers trnH2 and psbA demonstrated the potential usefulness of this plastid region [43], inducing us to design specific primers for *V. speciosa* using the plastidial genome of the species (accession number: SRX2844191; [44]); VS-trnH^GUG^2 (5′-TGGATCCACAATCCATTGC-3′) and VS-psbA2 (5′-CGTAATGCTCATAACTTCCCTCT-3′). PCR reactions were performed in 25 µL using KAPA2G Robust HotStart ReadyMix PCR kit (Kapa Biosystems, Roche Holding AG, Basel, Switzerland) and containing 12.5 μL of 5X KAPA2G Robust HotStart ReadyMix (1 U of KAPA2G Robust HotStart DNA Polymerase, 0.2 mM of ecah dNTP at 1X, 2 mM of MgCl_2_ at 1X), 0.5 mM of each primer, and 50 ng of DNA. The amplification program consisted of a first 3 min step at 95 °C followed by 30 cycles of 95 °C for 30 s, 55 °C for 30 s, and 72 °C for 30 s, and a final extension step at 72 °C for 5 min. Automated sequencing of the purified PCR products was performed using VS-trnH^GUG^2 primer on a 3100-Avant Genetic Analyzer (Applied Biosystems, Foster City, CA, USA) in the “Centro de Instrumentación Científica” of the University of Granada (Granada, Spain).

We used the *gapCp* gene as nuclear marker, which has been used successfully in various phylogenetic studies of Hymenophyllaceae species, including the genus *Vandenboschia*, and has shown variation at infraspecific levels (e.g., [45,46,47]). Due to the tetraploid nature of *V. speciosa* ([48]; although, flow cytometry data from Irish populations suggest that there could be a mix of cytotypes; [49]), the nuclear *gapCp* gene was only amplified in gametophytes (diploids) to avoid methodological challenges related to intra-individual gene copy number increase. In total, 150 *V. speciosa* gametophytes from 36 populations and one sporophyte of each *V. boschiana* and *V. davallioides* accession were analysed for the nuclear *gapCp* gene. PCR reaction conditions were the same than those for the plastid region except for the addition of 5 µL NZYTaq 5X Optimizer Solution (NZYTech, Lisbon, Portugal). The primers used for PCR amplification, GapC-7FA and GapC-BR-1, and cycling parameters followed Ebihara et al. [45]. Purified PCR products were ligated into the pSC-A-amp/kan vector of the StrataClone PCR Cloning Kit (StrataGene, Agilent technologies, Santa Clara, CA, USA) and cloned in StrataClone SoloPack competent cells (StrataGene), following the manufacturer’s recommendations. Between three and five recombinant clones were sequenced per individual, using the generic primer M13F at the “Centro de Instrumentación Científica” of the University of Granada (Granada, Spain).

For both ptDNA and nuclear *gapCp*, sequences were edited and aligned, using the Clustal algorithm, in the alignment editor BIOEDIT v7.0.5.3 [50], and then adjusted by eye. 

### 2.3. Establishment of gapCp Variant Homology by Phylogenetic Analysis

By visual inspection of the *gapCp* alignment, we detected a set of nucleotide positions that differentiate two types of sequences in *V. speciosa*. To establish the homology relationship between these variants, we carried out a maximum likelihood (ML) phylogenetic analysis, including the *V. speciosa* sequences, those established by us for *V. boschiana* and *V. davallioi*des, and the *gapCp* sequences in GenBank for *V. radicans* group and *V. auriculata* (subgenus *Lacosteopsis*; as outgroup species) ([45]; accession numbers: AB196370-AB196419). Inter-copy recombinant and single-point mutation sequences were detected with DNAsp v5.10 [51] and removed from alignment [52], which finally included 491 sequences in total. For phylogeny reconstruction, we used haplotypes instead of all sequences; haplotypes were detected with Arlequin v3.5.2.2 [53]. Phylogenetic analysis was conducted with PhyML v3.0 [54] through the PhyML web server (http://www.atgc-montpellier.fr/phyml-sms/, accessed on 18 February 2022), with the nucleotide substitution model automatically selected by the Smart Model Selection tool integrated in the PhyML web server [55], and the tree searching starting with five random trees obtained by BIONJ algorithm [56] and SPR as branch swapping method. Branch supports were assessed by the Shimodaira–Hasegawa-like (SHL) implementation of the approximate likelihood ratio test [57]. Because the two *gapCp* variants were homoeologs (called gapCp-572 and gapCp-575, see Section 3), we independently considered both copies in the subsequent analyses and only the largest intra-copy nonrecombining portion (detected with DNAsp).

### 2.4. Genetic Diversity and Structure

Population and regional genetic diversities were assessed by the number of haplotypes (ha), haplotype diversity (Hd), and nucleotide diversity (π), calculated with Arlequin v3.5.2.2 [53] for both the ptDNA and the two copies of *gapCp*. For ptDNA and mixed populations (gametophytes and sporophytes), diversity indices were calculated considering all individuals together (including both life cycle phases), and for the independent phases separately.

The distribution of genetic variability between generations (gametophyte and sporophyte) was evaluated for ptDNA using an analysis of molecular variance (AMOVA; [58]) and tested with a permutation test (10,000 permutations) with Arlequin. Hierarchical AMOVAs were also conducted to quantify the proportion of total genetic variance explained by the difference between the 11 geographical regions and between populations within regions, for all molecular markers. In addition, two more AMOVAs were performed for ptDNA, considering the geographical distribution of the haplotypes and the results of the network analysis (see Section 3). Thus, we considered the highest level of population grouping to be called the “evolutionary unit”. The AMOVAs were made to test the differentiation between evolutionary units, and they included, first, two evolutionary units (Northern and Southern) and the Cantabrian region; and later without taking into account the Cantabrian region. The Cantabrian region was considered an independent unit due to the high admixture of haplotypes from the northern and southern evolutionary units. 

Haplotype networks were reconstructed for all molecular markers (ptDNA, gapCp-572, and gapCp-575) following the statistical parsimony method as implemented in TCS v1.21 [59].

### 2.5. Haplotype Phylogeny and Dating

Phylogenetic relationships among ptDNA haplotypes of *V. speciosa* and the outgroup species were inferred using Bayesian inference (BI), with MrBayes v3.1.2 [60], and the best-fit nucleotide substitution model (HKY), as implemented in MrModeltest version 2.3 [61] and considering the Akaike’s information criterion [62]. The analysis was based on 2 million generations with 4 simultaneous runs (16 Markov chain Monte Carlo chains) starting from random trees that were sampled every 100 generations. Tracer v1.7 [63] was used to check the stationary of the runs and the convergence between runs. The initial 25% of the trees that resulted were discarded as burn-in, and the remaining trees were used to build 50% majority rule consensus trees. 

To relate genetic differentiation found among ptDNA haplotypes to Neogene–Quaternary events, we estimated divergence times using BEAST2 package [64]. We followed a two-step strategy for tree calibration, because we could not establish specific dates for the few known fossils (the oldest considered to be from the Upper Miocene; see [65]). An initial analysis, to estimate the divergence time of *V. speciosa* lineage, was implemented with sequences of the *rbc*L gene (due to the higher availability of sequences in the nucleotide database), all taken from the GenBank database (accession numbers on the resulting tree; see Section 3) except for sequences from the *V. boschiana* collections and two individuals of *V. speciosa* (from CID, Azores, and LIM, Ireland, populations) generated by us. The two sequences of *V. speciosa* obtained from GenBank are from individuals of populations in France (accession number: Y09201) and Andalusia (Spain, COQ population; accession number: NC041000). The sequences were amplified using the same PCR conditions described for the *trn*H-*psb*A region and the primers designed by us, using the plastidial genome of the species (accession number: SRX2844191; [44]), VSrbcL1F (5′-ATGTCACCACAAACCGAGACTAAAAC-3′; modified from Levin [66], 2003) and VSrbL1361R (5′-TCAGGACTCCACTTACTAGCGTCACG-3′). The analysis included 37 *rbc*L sequences in total, four of *V. speciosa*, 23 of other 15 *Vandenboschia* species, and 10 sequences of 8 external species to *Vandenboschia* selected as representative of the main lineages of trichomanoids [(*Crepidomanes* (3 species), *Trichomanes ankersii*, *Abrodictyum elongatum*)] and *Hymenophyllum* (3 species) according to Schuettpelz and Pryer [67]. Phylogeny was calibrated employing the dates proposed by Testo and Sundue [68] by constricting the origins for the following: Hymenophyllales (277.71–235.24 million years ago (Ma), mean: 242.93 Ma); *Hymenophyllum* (185.13–136.93 Ma, mean: 146.86 Ma; used by us as the most external taxon); all trichomanoid genera (235.74–223.44 Ma, mean: 232.76 Ma); the common ancestor of genera *Abrodictyum*, *Cephalomanes*, and *Trichomanes* (210.2–182.67 Ma, mean: 201.98 Ma); the common ancestor of *Vandenboschia* and *Crepidomanes* (171.78–143.38 Ma, mean: 156.24 Ma); the common ancestor to *Crepidomanes* (136.59–129.04 Ma, mean: 133.07 Ma); and the common ancestor to *Vandenboschia* (120.57–96.43 Ma, mean: 104.3 Ma). The partitioned (for codon positions) .xml file was made up in BEAUti v2 [64] by using a GTR+G+I substitution model, selected by jModelTest2 [69], an uncorrelated lognormal relaxed-clock model [70], after rejecting the strict molecular clock with the software BASEML of the PAML package [71], and a calibrated Yule model as the tree prior. BEAST v2.5.2 [72] was launched with 30 million generations sampling one tree and parameters every 5000 generations. Tracer v1.7 was used to check chain convergence and effective sampling size of the parameters. The maximum clade credibility tree (MCC) summarizing the estimated mean age and the 95% confidence intervals from post-burn-in (10%) trees was calculated with TreeAnnotator v2.5.2 [64]. The resulting MCC tree was visualized in FigTree v1.4.2 (http://tree.bio.ed.ac.uk/software/figtree/, accessed on 23 November 2020).

The second analysis concerned the *trn*H-*psb*A haplotypes of *V. speciosa*, using the sequences of five species of *Vandenboschia* (three sequences taken from Genbank, accession numbers on the tree in the Section 3, and two generated by us of *V. boschiana* and one of *V. davallioides*) as outgroup. Two calibration points were defined using the dates from the first analysis (indicated on the resulting tree; see Section 3). One for the common ancestor of the *V. davallioides* group (including *V. boschiana*, *V. davallioides*, *V. kalamocarpa*, *V. nipponica*, *V. speciosa*, and *V. subclathrata*), and another for the common ancestor of the same taxa except *V. davallioides*. BEAST package was used following the same procedure described above, but using the HKY substitution model, and the coalescent constant population model as tree prior. 

### 2.6. Demographic Analyses

The neutrality tests Fu’s *F* [73] and Tajima’s *D* [74] were used to detect possible historical demographic processes (expansion or contraction), using Arlequin. Both tests were performed considering geographical regions and evolutionary units, and with each molecular marker. The level of significance of both statistics was calculated by 10,000 simulated samples. In addition, ptDNA sequences were used to test for evidence of population size fluctuations within *V. speciosa* evolutionary units and also within Cantabrian range by constructing Bayesian skyline plots with BEAST (BSP; [75]). Analyses were performed assuming a strict clock model with a fixed clock rate inferred from the infraspecific BEAST analysis described earlier (1.08 × 10^−3^ s/s/y), a coalescent Bayesian skyline prior as a tree model with five groups of coalescent intervals, and the HKY model with empirical frequencies and the transition–transversion parameter (kappa) fixed to 4.3 according to the selected model. Additionally, we included a root height prior for each analysis, with a normal distribution, derived from the infraspecific dating analysis (mean 5.2 Ma, SD = 2.5). Analyses were run for 20 million generations and 2000 as sample frequency. We used Tracer to examine the trace files for convergence and plots after discarding the burn-in (10%). 

To reconstruct the historical migration routes in *V. speciosa* and locations of internal nodes of the haplotype phylogeny, ptDNA sequences were analysed using the marginal approximation of the structured coalescent implemented in BEAST2 package MASCOT v1.2.2 (Marginal Approximation of the Structured COalescenT; [76]). We defined 3 areas based on the evolutionary unit structuring: North, South, and Cantabrian. Prior substitution model was as performed in the BSP analysis. A lognormal distribution was used as prior for both the effective population size (Ne; M = 0 and S = 1) and the clock rate (M = 1.08 × 10^−3^, in the real space, S = 0.25), following the suggestion in “Taming the Beast” (https://taming-the-beast.org, accessed on 20 December 2020; [77]). MCMC analysis was run for 50 million generations, sampling every 5000 generations. The log file was analysed with Tracer to confirm adequate sample size and to determine the migration rates and Ne estimates. TreeAnnotator was used to summarize the trees in a MCC tree after discarding the first 10% as burn-in. FigTree was used to visualize the MCC tree.

### 2.7. Species Distribution Modelling

To identify past refugia and future distribution areas for *V. speciosa*, we evaluated the potential range of the species, considering the two phases of the life cycle both together and separately, under past, current, and future conditions with species distribution modelling (SDM). For environmental data, we used 19 BIOCLIM variables at a resolution of 2.5 arcminutes (c. 5 km). Past and current climate data were available from the WorldClim database (www.worldclim.org, accessed on 15 July 2017; [78]) and included data for the current-day period (1950–2000), the last glacial maximum (LGM; c. 21 ka) simulated by CCSM model (the community climate system model), and for the last interglacial period (LIG; c. 120 ka). In addition, we made predictions for future climatic conditions in 2080 for the most impacting IPCC’s climate scenario: RCP8.5 [79] available through the CCAFS Climate portal (www.ccafs-climate.org, accessed on 15 July 2017). Highly correlated variables (Pearson’s R ≥ 0.8) were reduced to eight uncorrelated variables (Table S2) used as predictors to calibrate the distribution models. Species occurrence data is a collection of references in databases—the Global Biodiversity Information Facility data portal (http://www.gbif.org/, accessed on 15 July 2017), the Biodiversity databank of the Canary Islands (http://www.biodiversidadcanarias.es/atlantis/common/index.jsf, accessed on 16 June 2013), and the Azores Biodiversity databank (http://www.atlantis.angra.uac.pt/atlantis/common/index.jsf, accessed on 1 February 2014)—the literature [36,80,81,82,83,84,85,86], and our own field records. A total of 1548 presence records (1066 for gametophyte and 482 for sporophyte) of *V. speciosa* were finally compiled (Figure 1). To perform the SDM, we applied maximum entropy modelling implemented in the software package MaxEnt 3.4.1 [87]. Models were generated using cross-validation of five replicate runs. Model performance was assessed on the basis of the area under the receiver operating characteristic curve (AUC). The contribution of each predictor variable in the model was analysed by the permutation importance and percent contribution coefficients (Table S2). A final reduced model including the most important variables [88], the mean diurnal range and the minimum temperature of the coldest month, was finally computed.

## 3. Results

### 3.1. PtDNA and Nuclear Marker Characteristics

The *trn*H-*psb*A sequences of *V. speciosa* showed eight polymorphic nucleotide positions among them. In *V. speciosa*, we detected two types of *gapCp* sequences, which we called gapCp-572 and gapCp-575 (572 bp and 575 bp in length, respectively). These differed by 20 inter-copy variable positions (but fixed within each copy) and 5 indels. The number of polymorphic positions in the final alignment was 344 and the number of indels was 17. ML phylogenetic analysis showed different relationships for both *gapCp* copies (Figure S1), since gapCp-575 was closely related to the Ebihara’s type-C copy (*V. birmanica*) and gapCp-572 was related to the Ebihara’s type-A and type-B copies (*V. kalamocarpa* and *V. nipponica*, respectively), and one of the two sequence types found in *V. boschiana* (type-I in Figure S1). The latter species showed a second type of sequences related to the *V. davallioides* lineage (type-II in Figure S1). The two types of sequences found in *V. boschiana* were detected in both individuals analysed. Independent alignments for *V. speciosa gapCp* copies, including only the largest intra-copy nonrecombining portion, were 80 bp and 92 bp in length and included 23 and 35 polymorphic sites (gapCp-572 and gapCp-575, respectively). 

### 3.2. Genetic Diversity and Structure

For ptDNA, gapCp-572, and gapCp-575, the total number of haplotypes found were nine, 28 and 43, respectively. The results for the diversity indices are shown in Table 1. At the population level, the mean diversity values for the ptDNA were *Hd* = 0.695 and *π* = 0.00312, while for gapCp-572 were *Hd* = 0.273 and *π* = 0.00368, and *Hd* = 0.323 and *π* = 0.00413 for gapCp-575. At the geographical region level, the most diverse regions were the following: the Basque Country for the ptDNA; Vosges du Nord and Italy for gapCp-572; and Italy for gapCp-575 (Table 1). 

**Figure 2:**
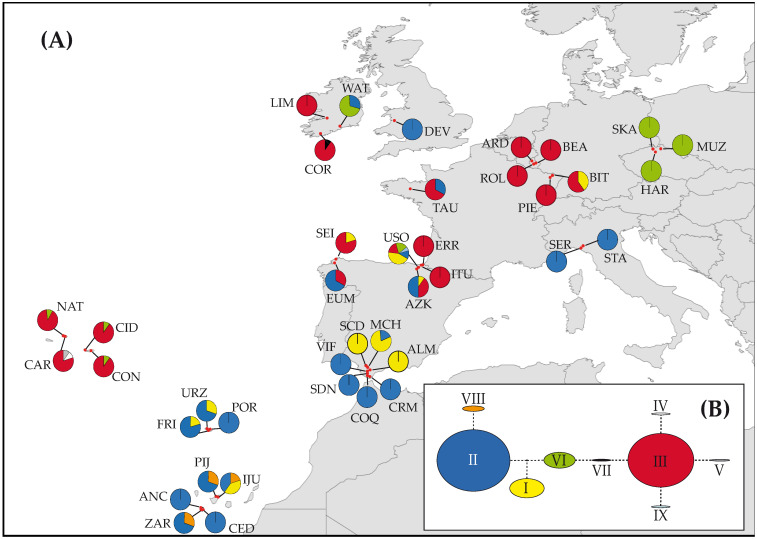
Distribution and network of the cpDNA haplotypes detected in the populations sampled of *Vandenboschia speciosa*. (**A**) Geographical distribution of the cpDNA haplotypes among the 40 populations sampled (see Table S1 for population code); pie charts indicate haplotype frequency. (**B**) Inferred cpDNA network, following the statistical parsimony method, with TCS; haplotypes are denoted by Roman numerals, and circle sizes are proportional to the haplotype frequencies.

The representation on a map of the ptDNA haplotype frequencies and distributions suggests a geographical structuring (Figure 2A). Haplotypes H-II and H-III are the most frequent and widespread, showing a mainly differentiated distribution. Haplotype H-II is dominant into the southern regions but also appears in the northern Iberian Peninsula as well as in Brittany, Ireland, and Wales. On the other hand, H-III dominates in the central and northern geographical regions and coexists with H-II in the northern Iberian Peninsula. Other haplotypes distributed mainly in the southern regions are H-I, though scantily represented in the northern Iberian Peninsula and Luxemburg, whereas H-VIII only detected in the Canary Islands. Finally, other central and northern haplotypes are the following: H-VI and the private and single individual haplotypes H-IV and H-V (Azores), H-VII (Ireland), and H-IX (Basque Country). Plastid–DNA network analysis resulted in the network shown in Figure 2B. According to the geographical distribution of ptDNA haplotypes and their relationships in the network, we defined two supra-regional population groupings that we termed “evolutionary units” (North: ARD-BEA-BIT-CAR-CID-CON-COR-DEV-HAR-LIM-MUZ-NAT-PIE-ROL-SKA-TAU-WAT; South: ALM-ANC-CED-COQ-CRM-IJU-FRI-MCH-PIJ-POR-SCD-SDN-SER-STA-URZ-VIF-ZAR). Populations in the Cantabrian region (AZK-ERR-EUM-ITU-SEI-USO) were considered as belonging to an independent region due to the high admixture of haplotypes from the northern and southern evolutionary units.

For *gapCp*, each copy has a main haplotype which is present along the 36 sampled populations. Of the 27 and 42 minority haplotypes found for gapCp-572 and gapCp-575, respectively, most were single individual haplotypes. Network analyses resulted in only one star-like-shaped haplogroup for each *gapCp* copy (Figure S2). No *gapCp* copy showed apparent haplotype geographical structuring (data not shown). 

AMOVA analyses of ptDNA sequences showed no genetic differentiation between sporophyte and gametophyte (*F*_CT_ = 0.011, *p* = 0.215), indicating that the genetic variation resides mainly among populations (68.95%, *p* < 0.001) rather than between generations (Table 2). This result induced us to combine gametophyte and sporophyte data in all remaining analyses, and not to consider the two phases separately. When the 11 geographical regions were considered, almost 39% (*p* < 0.001) of variation was between regions (Table 2). Considering supra-regional groupings, when we excluded the Cantabrian region, a clear differentiation between the northern and southern units become evident (*F*_CT_ = 0.51, 50.77% of variation, *p* < 0.001; Table 2).

AMOVA analyses of *gapCp* sequences and 11 regions revealed that almost all the genetic variation in both gapCp-572 and gapCp-575 copies resides within populations (99.63% and 99.13%, respectively; Table 2). 

### 3.3. PtDNA Haplotype Phylogeny and Dating

The phylogenetic tree resulting from the Bayesian analysis (Figure S3) showed how the haplotypes of *V. speciosa* formed a group (posterior probability, pp = 1), which includes the haplotype found in *V. boschiana*. The relationships between *V. speciosa* haplotypes agree with those found in the ptDNA network: H-III, H-IV, H-V, H-VII, and H-IX form a clade (pp = 0.82); haplotypes H-II and H-VIII group together; and relationships of haplotypes H-I and H-VI remain ambiguous. 

**Figure 3:**
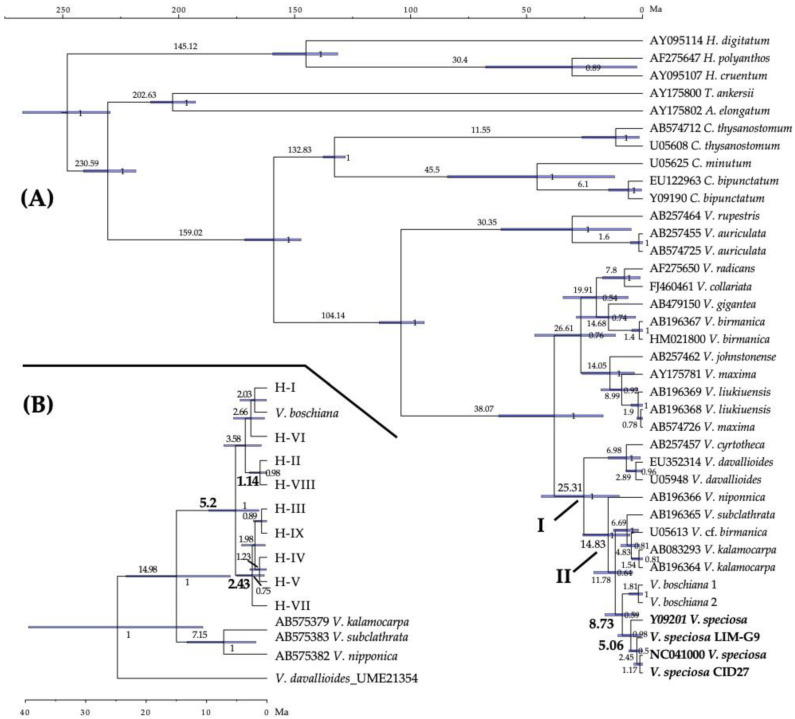
Maximum clade credibility trees summarizing the estimated mean ages and the 95% confidence intervals obtained with BEAST. (**A**) Diversification–time estimates for the genus *Vandenboschia* and outgroups using the *rbc*L dataset; sequences for *Vandenboschia speciosa* are marked in bold text; the bold Roman numerals indicate the dates used to calibrate the *trn*H-*psb*A haplotype phylogeny. (**B**) Time-calibrated phylogeny for the *Vandenboschia speciosa trn*H-*psb*A haplotypes and outgroup species. Numbers above branches are the mean divergence ages (in million years ago; Ma) for each node; the ages for the key nodes of *V. speciosa* are shown in bold; blue bars represent 95% highest posterior density intervals for each node; numbers after nodes are BEAST posterior probabilities (only pp > 0.5 are shown); accession numbers of sequences taken from GenBank are shown before the species name; the time scale is printed in million years ago (Ma).

The first step in the divergence–time estimates resulted in the beginning of the diversification of *V. davalloides* group 25.31 Ma (95% HPD: 9.99–43.6), in the late Oligocene, and that of the *V. nipponica*-*kalamocarpa* group 14.83 Ma (95% HPD: 5.45–25.74) during the Middle Miocene (nodes I and II in Figure 3A). In contrast to the *trn*H-*psb*A region, the *V. speciosa rbc*L sequences form a monophyletic group that is sister to the *V. boschiana* sequences and whose diversification began 8.73 Ma (95% HPD: 2.17–16.21), during the mid-Tortonian (Figure 3A). Using these dating evaluations, as secondary calibration points (nodes I and II in Figure 3A), we dated the *trn*H-*psb*A haplotype nodes of *V. speciosa* (Figure 3B). The *trn*H-*psb*A analysis suggests the following: that the start of the diversification of the haplotypes occurred 5.2 Ma (95% HPD: 1.34–9.64), coinciding with the estimated date of diversification of the *rbc*L sequences (5.06 Ma; Figure 3A); that diversification of the haplogroup including H-III began in the Pliocene–Pleistocene transition (2.43 Ma; 95% HPD: 0.44–4.98); and that H-VIII diverged from H-II 1.14 Ma (95% HPD: 0.01–2.91) (Figure 3B).

### 3.4. Demographic Analysis of V. speciosa

For ptDNA, the neutrality tests turned out to be non-significant (Table S3); however, negative values close to significance were registered for the Azores region. At the evolutionary unit level, the northern unit was the only one that had negative values (not significant). For nuclear markers, all tests resulted in negative and significant values for all geographical regions and evolutionary units.

**Figure 4:**
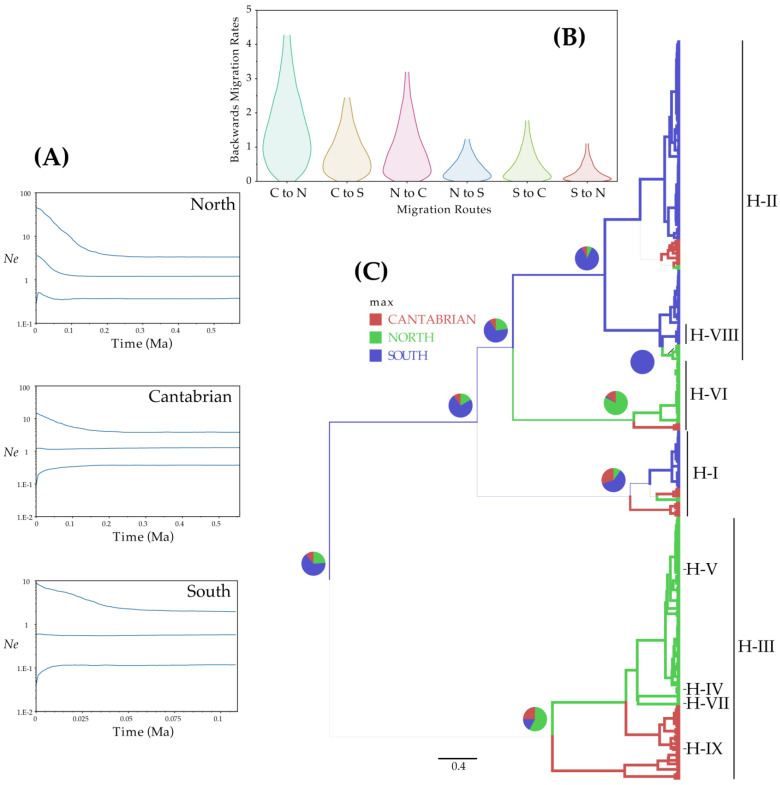
Demographic analyses based on coalescence considering the supra-regional groupings of *Vandenboschia speciosa* (evolutionary units and the Cantabrian Cornice), inferred from ptDNA and implemented with BEAST. (**A**) Bayesian skyline plots depicting changes in effective population size (*N*_e_) as a function of time (in million years ago, Ma); in each plot, the centre line is the median estimate, and the upper and lower lines delimit the highest posterior density (HPD) 95% confidence intervals for *N*_e_. (**B**) Violin plot of the inferred backwards migration rates between the different regions (C, N, and S: Cantabrian Cornice, Northern, and Southern evolutionary units, respectively), using the marginal approximation of the structured coalescent (MASCOT) as a population prior. (**C**) Maximum clade credibility tree showing the inferred root regions for the ptDNA haplotype, using MASCOT as a population prior; the pie charts show the inferred probability of the root being in any of the three regions.

The BSP analyses found evidence for range expansion only for the northern evolutionary unit, this starting from 100 to 80 thousand years ago (Ka; beginning of the Würm glaciation) (Figure 4A).

The structured coalescent approach identified migrations occurring mainly between the northern and Cantabrian regions (Figure 4B), with a greater proportion of individuals from the Cantabrian region coming from the north (mean: 1.76); although, a relatively high migration rate was detected in the opposite direction (mean: 1.12). The migration rate from the south to the Cantabrian region was close to unity (mean: 0.96), while the rest of the rates proved extremely low (means < 0.6). On the other hand, MASCOT analysis yielded in the ancestral locations of *V. speciosa* lineages displayed in Figure 4C. The South was the root state with moderate probability (0.65), where haplotypes H-I and H-II originated (probabilities: 0.6 and 0.86, respectively) and from which they migrated into the Cantabrian and northern regions. The haplotype H-VIII, only detected in the Canaries, originated there from H-II. Haplotype H-VI originated in the north (probability: 0.82) and dispersed into the Cantabrian Cornice. Finally, the origin of haplotype H-III was resolved as the north with little more than the 50% probability (0.58), and the remaining haplotypes (exclusive to different regions) originated from in situ H-III. 

### 3.5. Species Distribution Modelling 

**Figure 5:**
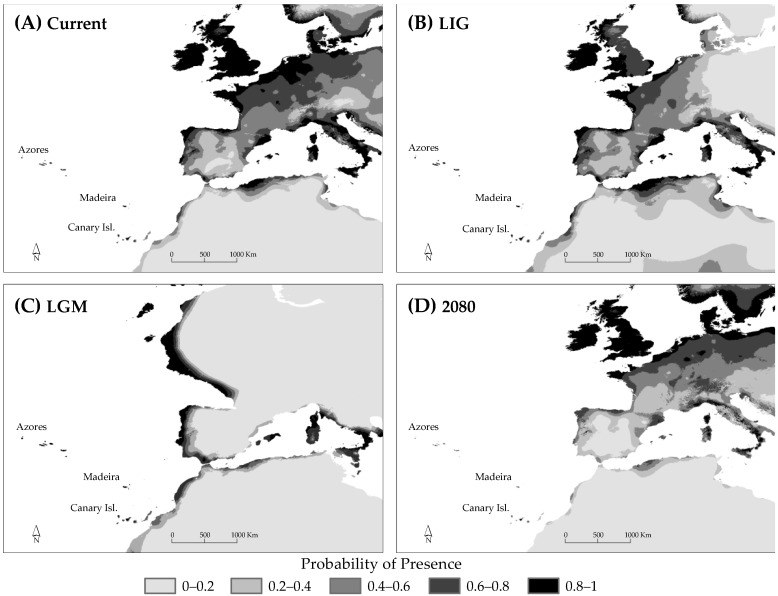
Maps of predicted environmental suitability for *Vandenboschia speciosa* using the maximum entropy algorithm and the community climate system model (CCSM), as implemented in MaxEnt. Map showing the projections for current (**A**), last interglacial (LIG, *c*. 120–140 ka) (**B**), last glacial maximum (LGM, *c*. 21 kya) (**C**), and future (2080) (**D**), are shown for the species as a whole. The probability of presence is shown as continuous values from the threshold (defined as maximum sensitivity plus specificity) to 1.

For all models, the AUC values were high (minimum value of AUC = 0.962). For the species (gametophyte and sporophyte together), the MaxEnt current and LIG predictions showed regions of suitable habitats that coincided largely with the current distribution, with additional areas of the distribution range on the European Atlantic coasts further north and more widely along the Mediterranean sea, where the species is currently absent (Figure 5A,B). Palaeodistribution modelling for LGM suggested a strong contraction of the suitable habitats in Northern and Central Europe (Figure 5C). According to LGM output, refugia were located in Macaronesia, the European Atlantic coast, and a few regions on the Mediterranean coastline. The Atlantic coastal strip of the Iberian Peninsula, from Galicia to the south, appeared as a continuous zone of high suitability. This continuity was not found in the LIG predictions (Figure 5B). The future projections, using the RCP8.5 scenario, suggested a partial reduction in the suitable habitats of the species on the coasts of Portugal, northern Iberian Peninsula, and Macaronesian islands, along with an increase in suitable habitats north of the European Atlantic coast (Figure 5D). Independent analyses for gametophyte and sporophyte resulted in that the gametophyte presented a wider distribution than the sporophyte (for past, present, and future), especially towards the north and the east of the continent (Figure S4).

## 4. Discussion

### 4.1. Absence of Genetic Structure between the Sporophytic and Gametophytic Phases of V. speciosa

*Vandenboschia speciosa* is one of the few fern species with a long-lived gametophytic phase [89]. Gametophytes differ from sporophytes in ecological tolerances and dense clonal populations can result (by producing gemmae quite effective as dispersers over short distances) without sporophytes in continental environments, beyond the oceanic environments to which sporophytes are limited. These differential characteristics between gametophytes and sporophytes point to the possible isolation and genetic differentiation of gametophytic populations. However, our AMOVA results showed no structuring of plastid genetic diversity between gametophytes and sporophytes (Table 2); as Rumsey et al. [40] observed using allozyme banding phenotypes in three sites of southwestern Scotland. This result supports the suggestion of those authors that the gametophyte stage, harbouring all of the genetic variability observed in the analysed sporophytes, can be regarded as a “seed-bank” and a genetic reservoir for the species [40].

### 4.2. Tertiary Origin of V. speciosa

Our results show a Tertiary origin for *V. speciosa* at the beginning of the Late Miocene, when it diverged from *V. boschiana*, placing it as part of the European palaeotropical flora. This dating agrees well with the oldest known fossils of the species in Georgia (Upper Miocene; [65]). 

According to the chromosomal counts of Manton [48] *V. speciosa* is a tetraploid species whose origin is not clear. Ebihara et al. have suggested that it could be an allotetraploid (unpublished data; suggested in [90]). Ní Dhúill [49] found variation in cytotypes (diploid, triploid, and tetraploid sporophytes, but never haploid gametophytes) in several Irish populations using flow cytometry, suggesting that the origin of the species could be more complicated than previously thought. Rumsey, considering the morphological and cytotype variation, raises the possibility that *V. speciosa* has had polytopic origins and that there may be more than one cryptic taxon (pers. com.). The two variants we detected in *V. speciosa* for the *gapCp* gene (gapCp-572 and gapCp-575, with differential additivity to the *gapCp* of species of well-differentiated clade (Figure S1), support the allopolyploid origin of *V. speciosa* from a species related to the *V. niponnica*-*kalamocarpa* group lineage and from a species related to the *V. birmanica* lineage. However, the fact that all the analysed plastidial sequences of *V. speciosa* (both for the *rbc*L gene, Figure 3A, and for the *trn*H-*psb*A region, Figure S3) share a common ancestor, and the fact that we did not detect any plastidial sequence related to *V. birmanica* among all analysed populations and individuals, suggests the rejection of the hypothesis of the species’ polytopic origins and cryptic taxa, unless we assume that the species related to *V. niponnica-kalamocarpa* group always acted as the female parent. Our *gapCp* phylogeny also shows additivity for sequences obtained from *V. boschiana*, with one set of sequences included in the *V. nipponnica*-*kalamocarpa* group and another group related to *V. davalloides* sequences, also suggesting a hybrid origin for this species endemic to eastern North America and for which diploid and tetraploid fertile sporophytes are known. More detailed studies are needed to identify whether hybridisation in *V. boschiana* is recent or ancestral hybridisation. The inclusion of the *trn*H-*psb*A sequence of *V. boschiana* among the haplotypes of *V. speciosa* could suggest the possible involvement of this species in the origin of *V. speciosa* (Figure S3). However, considering the reciprocal monophyly between both species in the phylogeny with the *rbc*L gene (Figure 3A), the most likely explanation seems to be due to the paucity of phylogenetic information in *trnH-psbA* (as is also the case of *V. kalamocarpa* and *V. subclathrata*) and that both lineages diverged from a common ancestor probably before hybridisation occurred in the *V. speciosa* lineage. The fact that the *V. speciosa gapCp* sequences (the gapCp-572 variant; Figure S1) do not form a sister group or intermingle with *V. boschiana* sequences supports the latter idea, as the opposite would be expected due to the slower lineage sorting expected for nuclear sequences than plastid sequences because of their larger effective population size and, especially, to the complete additivity of the parental genes expected for an allopolyploid species, mainly when the parental species belong to lineages as differentiated as those involved in the origin of *V. speciosa* so that homoeologs chromosomes are unable to pair with each other [91].

The beginning of the *V. niponnica-kalamocarpa* group lineage dates back to the late Oligocene (25.3 Ma, 95% HPD: 9.99–43.6; Figure 3A), while the time of diversification and appearance of the main lineages within the group was concentrated between 14.8 and 8.7 Ma (95% HPDs: 5.45–25.74 and 2.17–16.21, respetively; Figure 3A), coinciding with the onset of the Miocene cooling after the mid-Miocene climatic optimum [92]. This is consistent with a wide distribution of the ancestor of this group during the late Oligocene warming [93] and the subsequent range fragmentation and lineage diversification triggered by the Miocene cooling. A possible ancestor could be *Trichomanes sacci* (=*T.* cf. *radicans*), a *Vandenboschia* species that inhabited Eastern Europe from the upper Oligocene to middle Miocene [65,94]. Currently *V. speciosa* is the only European representative of the genus and shows a strong geographical isolation from its closest relative *V. boschiana*, endemic to eastern North America. From middle–late Miocene (14–10 Ma), when both lineages diverged (Figure 3A), to the late Pliocene (3.5 Ma), the cooler and drier climatic conditions in Beringia (only land bridge between Asia and North America during this period) caused the warm–temperate groups were restricted to disjunct refugia in the south, resulting in vicariances [95] as could be that of *V. boschiana* and *V. speciosa*, and probably favouring contact between lineages in these refugia that may have facilitated hybridisation events, such as those observed in *V. speciosa*. In Europe, after the climatic cooling, the Arcto–Tertiary flora extended towards the south and towards the west, making retreat to the Paleotropical flora [2,96]. The fossil record of *V. speciosa* found in Eastern Europe and western Asia supports the past occurrence of the species in areas between Europe and East Asia; a scenario proposed also for *Davallia canariensis* [35].

### 4.3. Climate-Change-Driven Phylogeographical History of V. speciosa

The genetic structure detected for *V. speciosa* across its distribution range, with the plastidial marker *trn*H-*psb*A (Figure 2A), indicates the presence of two main evolutionary units with a differentiated northern and southern distribution that correlates basically with the biogeographical regions occupied by this fern (Eurosiberian, Mediterranean). The main haplotypes from both units coexist in the Cantabrian Cornice, and the southern H-II extends, infrequently, northwards along the European Atlantic coast. A similar pattern was discerned by Rumsey et al. [42] for the distribution of the only one polymorphic position detected by PCR-RFLP in the plastid *trn*L-F region. 

Our results suggest that this structuring was due to the action of pre-Pleistocene historic rather than biogeographical factors. These are consistent with: (1) long persistence, during Neogene, in independent refugia of the Eurosiberian Atlantic and Mediterranean coasts (considering Madeira and the Canary Islands as part of the Mediterranean Region) and independent evolution of gene pools, and the (2) north–south dispersals tracking the Quaternary glacial cycles. 

The initial differentiation of *V. speciosa* gene lineages during the Miocene–Pliocene transition (Figure 3) suggests that the strong aridification at the end of the Miocene, which culminated with the Messinian salinity crisis (MSC: 5.96–5.33 Ma; [97]), caused the contraction of the area, population fragmentation and isolation, and the North–South genetic differentiation of *V. speciosa*. The laurophyllous vegetation (with which *V. speciosa* is associated) was forced to retreat towards moist locations of the Mediterranean and Black Sea basins, and the Macaronesian archipelagos [98,99]. Moreover, the Atlantic influence on the western end of Europe probably buffered the sharp climate change, allowing the survival of *V. speciosa* and other relict Tertiary plants [11,98]. A similar pattern has been suggested for *Davallia canariensis* [35]. The establishment of the Mediterranean climate (3.2 Ma; [100]) probably favoured the increase in the differentiation between the Eurosiberian and Mediterranean populations, due to the imposed climate constraints for range expansion, especially on the Mediterranean populations. According to Benito Garzón and Sainz Ollero [101], *V. speciosa* is one of the Tertiary ferns that survived in Iberian and Macaronesian shelters to the change towards the Mediterranean climate. It is plausible that the Mediterranean climate greatly reduced the Mediterranean populations, generating strong isolation and lowered genetic diversity by genetic drift.

Unlike the Flora Lusitanica [102,103], our results do not support the idea that the northern populations originated from those of the south by post-glacial recolonization. The distribution model of genetic diversity shows the concentration of diversity along the Atlantic coast, with intermediate levels in most southern regions and higher levels in most northern regions (Table 1; Figure 2A). Moreover, the presence of private haplotypes in regions of the northern unit, such as the Azores and Ireland, suggests the long presence of *V. speciosa* in these areas. These private haplotypes are derived from the H-III haplotype (Figure 2B and Figure 4C), whose diversification started about Pliocene–Pleistocene transition (Figure 3B), supporting the presence of *V. speciosa* in these regions before the glaciations and its persistence during the Quaternary. 

### 4.4. The Cantabrian Cornice Could Act as a Tertiary Refugium and as a Suture Zone during the Quaternary

Our findings suggest the Cantabrian Cornice as a main refugium for *V. speciosa* during the Pliocene, in addition to the Pleistocene refugium traditionally accepted for ferns and other plants [10,25,31,102,103]. This area harbours an endemic haplotype and the highest haplotype diversity (for ptDNA; Figure 2A,B; Table 1), evidencing long-term occupation [24] and demographic stability (Figure 4A). 

On the other hand, the mixture of haplotypes from the south and the north on the Cantabrian coast also suggests a complementary hypothesis—that is, this region might have been a more recent secondary contact area between the two lineages. Our results imply that this contact happened by the southward expansion of the northern lineage (evidenced by BSP and MASCOT analyses; Figure 4A–C), in response to changes in habitat suitability caused by glacial cycles (SDM suggests a migration route during the last glacial period, by supporting the entire Iberian Atlantic coastal strip and also the French and Irish coasts, as almost continuous refugia during the LGM with high proportion of suitable habitats; Figure 5C). The southern lineage could have survived the glacial period along the entire Atlantic coastal strip of the Iberian Peninsula (Figure 5C) and may have separated from the Cantabrian Cornice during the post-glacial period (Figure 5B), when the Mediterranean climate returned along the entire western and southern Iberian coast. This would have left the species in small, favourable climatic pockets, as occurs at present, under the effect of strong genetic drift [29]. Rumsey et al. [41] has also suggested this coastal connection and its fragmentation by a process of aridification.

Finally, the MASCOT analysis also suggests migrations of H-I and H-II into northern regions (Figure 4B,C). Given the demographic stability of the southern and Cantabrian regions (Figure 4A; Table S3), these results might reflect long-distance dispersal events. 

### 4.5. Central and Northern Europe

One of the most intriguing biogeographical issues of *V. speciosa* concerns when, how, and from where the gametophyte reached its wide continental and Northern European distribution. Our SDM results show a strong effect of the glaciations on the populations of Central and Northern Europe (which would have disappeared), with northern suitable areas only in SW of Ireland and the French and Iberian Atlantic coasts (Figure 5C), supporting the hypothesis of post-glacial colonization—by long-distance dispersals—from southern and/or western refugia [37,40]; this opposes the hypothesis of spreading from more northerly Tertiary and peri-glacial refugia (not ruled out by the aforementioned authors). Moreover, the almost total absence of ptDNA diversity in the Central European populations (Luxembourg, the Vosges, and the Czech Republic) agrees with the post-glacial colonization (Table 1). However, our data do not determine exactly when these populations might have become established and thus do not support or refute the post-glacial hypsithermal period (c. 8.000–4.500 years. B.P.) suggested by Rumsey et al. [37,40].

The haplotypes we found strongly differentiate Czech Republic populations from those of Luxembourg and the Vosges (Figure 2A), suggesting at least two independent long-distance dispersal events. In the Czech Republic, we found only H-VI, suggesting Ireland and/or the Cantabrian Cornice as possible source areas. The presence of H-I in the Vosges connects this region with the southern refugia (Andalusia) or with the Cantabrian Cornice. Molecular markers with more resolution could help to increase the accuracy of these analyses. The *gapCp* sequences were not useful since they did not show any structure.

### 4.6. Macaronesian Archipelagos

Our results indicate the arrival of *V. speciosa* to Macaronesia through different dispersal events to different areas and at different times. The haplotype distribution shows the phylogeographical relationships between the Canary/Madeira archipelagos and Southern Iberia, and the relationship between the Azores and Northwestern Europe, suggesting different sources of colonization for these archipelagos. A similar pattern has been observed in *Asplenium hemionitis* [104]. In addition, the presence of two haplotypes in both Azores and the Canary/Madeira Islands that are shared with the rest of Europe (H-III and H-VI in Azores, and H-II and H-I in the Canaries and Madeira) suggest at least two independent dispersals to each of these island groups (each haplotype differs by several mutations making it unlikely for one of these haplotypes to arise from the other independently in Europe and each island group). Thus, the colonisation of Macaronesia by *V. speciosa* would add to the few reported cases of independent fern dispersals over such long distances shown in other parts of the world; such as the dispersals across the Tasman Sea between Australia and New Zealand of *Asplenium hookerianum* and *Asplenium flabellifolium* [23,105], or the multiple colonisations of the Hawaiian archipelago by *Asplenium adiantum-nigrum* [106].

The colonization of Macaronesia could have occurred during Plio–Pleistocene, as has been confirmed for relict angiosperms [107]. *Vandenboschia speciosa* reached the Canary Islands at least during the interglacial Donau-Günz, when the H-VIII haplotype, exclusive to the Canary Islands, diverged from H-II (1.14 Ma; Figure 3B), and probably from Madeira (with laurel forest since at least 1.8 Ma; [108]) or from the south of the Iberian Peninsula. Geological evidence indicates possible ancient land connections between the Canary Islands, Madeira, and Southern Iberia (by large volcanic seamounts; [109,110]), which are thought to have contributed substantially to the configuration of Macaronesian biota, perhaps accounting for the haplotype pattern observed for *V. speciosa* in the Macaronesia.

On the other hand, *V. speciosa* could have reached the Azores at the end of the Pliocene, before the glacial periods. Our data support the idea of a process of independent diversification of the four haplotypes derived from H-III (Figure 2B, Figure 3B and Figure S3), including the two exclusives of Azores, and that started 2.43 Ma (Figure 3B; external nodes in the H-III haplogroup lacks of support). This would place H-III in the Azores before that date, although a more recent origin of these private haplotypes cannot be ruled out. Góis-Marques et al. [111] concluded a Pleistocene–Holocene age for all plant fossils described in the Azores. *Vandenboschia speciosa* could have arrived to the Azores at Santa Maria Island (~6 Ma), the only island older than 1.5 Ma (reviewed by [108]). The presence in the Azores of haplotype H-VI suggests the immigration of the species from Cantabrian Cornice and/or from Ireland. 

### 4.7. Future Forecasts

In relation to the future, the projection of the SDM under 2080 climate predictions suggests a northward migration of *V. speciosa* and a high risk that the southern evolutionary unit could disappear (Figure 5D and Figure S4). The only areas that are maintained with steady potential are the UK and Ireland. This is consistent with the forecasts made for *Asplenium fontanum* and other ferns [27,112]. The expected reduction in the genetic diversity makes it necessary to intensify the conservation measures of the populations farther south.

## 5. Conclusions

Our results support the allopolyploid nature of *V. speciosa*, show a Tertiary origin for the species and the presence of two principal evolutionary units with a differentiated North (Eurosiberian Region) and South (Mediterranean Region) distribution. The strong aridification at the end of the Miocene that culminated with the Messinian salinity crisis is suggested to be the cause of this differentiation. The Cantabrian Cornice may have been a main refugium for *V. speciosa* during the Tertiary and the Pleistocene glaciations, but it could also have been a recent contact zone between the two lineages due to expansions of the area from the north towards the south and from the Cantabrian towards the north following the glacial–interglacial cycles. Current Central European populations appear to have derived from multiple post-glacial, long-distance dispersals. Our results indicate the arrival of *V. speciosa* to Macaronesia through different and repeated dispersal events and reveal the phylogeographical relationships between the Canary/Madeira archipelagos and Southern Iberia, as well as between the Azores and Northwestern Europe.

## Figures and Tables

**Table 1:** Information on number of haplotypes and diversity indices for *trn*H-*psb*A, gapCp-572, and gapCp-575 sequences in each population or region of *Vandenboschia speciosa* analysed in the present study.

	*psb*A-*trn*H				gapC-572				gapC-575			
Code	*ha*	*Priv*	*Hd*	*π*	*ha*	*Priv*	*Hd*	*π*	*ha*	*Priv*	*Hd*	*π*
Andalusia	2		0.425	0.0019	4	1	0.193	0.0087	8	4	0.340	0.0045
ALM	1		0	0	2		0.285	0.0124	2	1	0.182	0.0019
COQ	1		0	0	2	1	0.222	0.0096	3	1	0.416	0.0048
CRM	1		0	0								
MCH	2		0.355	0.0016	1		0	0	4	1	0.371	0.0058
SCD	1		0	0								
SDN	1		0	0								
VIF	1		0	0	2		0.250	0.0108	3	1	0.464	0.0054
Azores	4	2	0.233	0.0008	7	2	0.326	0.0153	8	3	0.371	0.0048
CAR	3	2	0.377	0.0009	2		0.200	0.0087	3	1	0.345	0.0055
CID	2		0.200	0.0009	3		0.524	0.0248	3	1	0.345	0.0039
CON	2		0.200	0.0009	3	1	0.464	0.0217	3	1	0.318	0.0036
NAT	2		0.200	0.0009	2	1	0.222	0.0097	3		0.472	0.0055
Basque Country	5	1	0.673	0.0030	6	1	0.331	0.0155	4	3	0.221	0.0025
AZK	3		0.644	0.0029	4	1	0.643	0.0326	2	1	0.286	0.0031
ERR	1		0	0	1		0	0	1		0	0
ITU	1		0	0	1		0	0	1		0	0
USO	5	1	0.767	0.0036	3		0.378	0.0174	3	2	0.464	0.0054
Canary Isl.	3	1	0.405	0.0013	5	3	0.192	0.0087	8	4	0.348	0.0041
ANC	1		0	0	1		0	0	2		0.333	0.0036
CED	1		0	0	2	1	0.286	0.0124	3	1	0.524	0.0062
IJU	3		0.711	0.0032	3	1	0.417	0.0193	2		0.222	0.0024
PIJ	2		0.467	0.0010	2	1	0.182	0.0079	3	1	0.345	0.0040
ZAR	2		0.467	0.0010	1		0	0	4	2	0.396	0.0047
Czech Republic	1		0	0	3	1	0.257	0.0116	6	5	0.447	0.0076
HAR	1		0	0	2	1	0.400	0.0174	1		0	0
MUZ	1		0	0	2		0.667	0.0290	5	4	0.786	0.0163
SKA	1		0	0	1		0	0	2	1	0.333	0.0036
Galicia	3		0.567	0.0025	4	1	0.249	0.0113	6	3	0.490	0.0060
EUM	2		0.533	0.0024	2		0.133	0.0058	6	3	0.778	0.0109
SEI	2		0.356	0.0016	3	1	0.464	0.0218	1		0	0
Ire-Wal-Bri *	4	1	0.611	0.0026	5	3	0.197	0.0089	10	7	0.379	0.0051
COR	2	1	0.200	0.0004	3	1	0.295	0.0134	2	1	0.222	0.0024
DEV	1		0	0	1		0	0	1		0	0
LIM	1		0	0	1		0	0	5	2	0.576	0.0072
TAU	2		0.533	0.0024	2	1	0.667	0.0290	1		0	0
WAT	2		0.467	0.0021	2	1	0.222	0.0097	5	4	0.667	0.0109
Italy	1		0	0	7	3	0.521	0.0261	6	4	0.515	0.0077
SER	1		0	0	5	2	0.667	0.0348	3	2	0.417	0.0072
STA	1		0	0	3	1	0.378	0.0174	4	2	0.643	0.0081
Luxembourg	1		0	0	4	2	0.331	0.0153	2	1	0.100	0.0011
ARD	1		0	0	3	1	0.524	0.0248	1		0	0
BEA	1		0	0	1		0	0	1		0	0
ROL	1		0	0	2	1	0.333	0.0145	2	1	0.333	0.0036
Madeira	2		0.351	0.0015	2		0.133	0.0058	1		0	0
FRI	2		0.467	0.0021	2		0.200	0.0087	1		0	0
POR	1		0	0	1		0	0	1		0	0
URZ	2		0.400	0.0018								
Vosges du Nord	2		0.356	0.0016	4	1	0.533	0.0261	4		0.350	0.0041
BIT	2		0.600	0.0027	2		0.400	0.0174	1		0	0
PIE	1		0	0	3	1	0.700	0.0348	4		0.714	0.0093

*—Ireland–Wales–Brittany; *ha*—number of haplotypes; *Hd*—haplotype diversity; *π*—nucleotide diversity; *Priv*—private haplotype.

**Table 2:** Hierarchical analysis of molecular variance (AMOVA).

Source of Variation	d.f	Sum of Squares	Variance Components	Percentage of Variation	Fixation Indices	*p*-Value *
***trn*****H**-***psb*****A**						
Among generations	1	4.042	0.00773 Va	1.084	*F*_CT_ = 0.011	0.21495
Among populations within generations	62	160.115	0.49222 Vb	68.954	*F*_SC_ = 0.7	**<0.001**
Within populations	245	52.400	0.21388 Vc	29.962	*F*_ST_ = 0.7	**<0.001**
Total	308	216.557	0.71383			
Among geographical regions	10	48.120	0.14279 Va	38.94	*F*_CT_ = 0.39	**<0.001**
Among populations within regions	29	25.525	0.09971 Vb	27.19	*F*_SC_ = 0.45	**<0.001**
Within populations	269	33.417	0.12423 Vc	33.87	*F*_ST_ = 0.66	**<0.001**
Total	308	107.061	0.36672			
Among evolutionary units (3 units)	2	34.062	0.16218 Va	39.47	*F*_CT_ = 0.39	**<0.001**
Among populations within units	37	39.583	0.12447 Vb	30.29	*F*_SC_ = 0.5	**<0.001**
Within populations	269	33.417	0.12423 Vc	30.23	*F*_ST_ = 0.7	**<0.001**
Total	308	107.061	0.41087			
Among evolutionary units (2 units)	1	31.022	0.23645 Va	50.77	*F*_CT_ = 0.51	**<0.001**
Among populations within units	32	33.948	0.13004 Vb	27.92	*F*_SC_ = 0.57	**<0.001**
Within populations	220	21.833	0.09924 Vc	21.31	*F*_ST_ = 0.78	**<0.001**
Total	253	86.803	0.46572			
**gapCp-572 copy**						
Among geographical regions	10	1.298	0.00037 Va	0.27	*F*_CT_ = 0.003	0.34851
Among populations within groups	25	3.066	−0.00218 Vb	0	*F*_SC_ = −0.016	0.72851
Within populations	235	32.544	0.13848 Vc	99.73	*F*_ST_ = −0.013	0.76386
Total	270	36.908	0.13667			
**gapCp-575 copy**						
Among regions (11 groups)	10	1.651	−0.00084 Va	−0.50	*F*_CT_ = −0.005	0.69554
Among populations within groups	24	4.518	0.00230 Vb	1.36	*F*_SC_ = 0.013	0.14871
Within populations	275	46.183	0.16794 Vc	99.14	*F*_ST_ = 0.008	0.25485
Total	309	52.352	0.1694			

*—Statistically significant values are indicated in bold text.

## Data Availability

All sequence data obtained in this study, ptDNA and *gapCp*, are available in the GenBank database (*rbc*L accession numbers: OM949956–OM949959; *trn*H-*psb*A accession numbers: OM949944–OM949955; *gapCp* accession numbers: ON033180–ON033620).

## Supplementary Materials

The following supporting information can be downloaded at: https://www.mdpi.com/article/10.3390/plants11070839/s1, Figure S1: Maximum likelihood tree for the *gapCp* sequences of *Vandenboschia speciosa* determined with PhyML, Figure S2: Statistical parsimony networks of the *gapCp* sequences, Figure S3: ptDNA haplotype phylogeny derived from the Bayesian inference with MrBAYES, Figure S4: Results of species distribution modelling for the sporophyte and gametophyte phases of the life cycle of *Vandenboschia speciosa* using the maximum entropy algorithm and the Community Climate System Model (CCSM), as implemented in MaxEnt, Table S1: Sampling details of *Vandenboschia speciosa* populations and outgroup species used in the present study, Table S2: Percentage contribution and permutation importance of selected model for the species distribution modelling (SDM), Table S3: Neutrality tests Fu’s *F* and Tajima’s *D* at the regional and supra-regional groupings.
