# Supplementary material for: Phylogeographical Analyses of a Relict Fern of Palaeotropical Flora (Vandenboschia speciosa): Distribution and Diversity Model in Relation to the Geological and Climate Events of the Late Miocene and Early Pliocene
Source: Plants (Basel). 2022 Mar 22;11(7):839. doi: 10.3390/plants11070839 (PMC9002575; doi:10.3390/plants11070839)
Supplement: Supplementary file 1 [file plants-11-00839-s001.zip › plants-1546277-supplementary.pdf]

SUPPLEMENTARY MATERIALS

Phylogeographical analyses of a relict fern of palaeotropical flora (*Vandenboschia speciosa*): distribution and diversity model in relation to the geological and climate events of the Late Miocene and Early Pliocene

Samira Ben-Menni Schuler, Hammadi Hamza, Gabriel Blanca, Ana Teresa Romero-García and Víctor N. Suárez-Santiago

The following Supporting information is available for this article:

Figure S1. Maximum likelihood tree for the *gapCp* sequences of *Vandenboschia speciosa* determined with PhyML.

Figure S2. Statistical parsimony networks of the *gapCp* sequences.

Figure S3. ptDNA haplotype phylogeny derived from the Bayesian inference with MrBAYES.

Figure S4. Results of species distribution modelling for the sporophyte and gametophyte phases of the life cycle of *Vandenboschia speciosa* using the maximum entropy algorithm and the Community Climate System Model (CCSM), as implemented in MaxEnt.

Table S1. Sampling details of *Vandenboschia speciosa* populations and outgroup species used in the present study.

Table S2. Percentage contribution and permutation importance of selected model for the species distribution modelling (SDM).

Table S3. Neutrality tests Fu's *F* and Tajima's *D* at the regional and supra-regional groupings.

Figure S1. Maximum likelihood tree for the 426 *gapCp* sequences of *Vandenboschia speciosa* and 15 of outgroups determined with PhyML; numbers above branches are support values from the Shimodaira-Hasegawa-Like implementation of the approximate likelihood-ratio test; the colours differentiate the two identified homoeolog copies; on the right are the names of the *V. speciosa* homoeologs, the names of the outgroup species, and the names of the sequences of Ebihara et al. (2005).

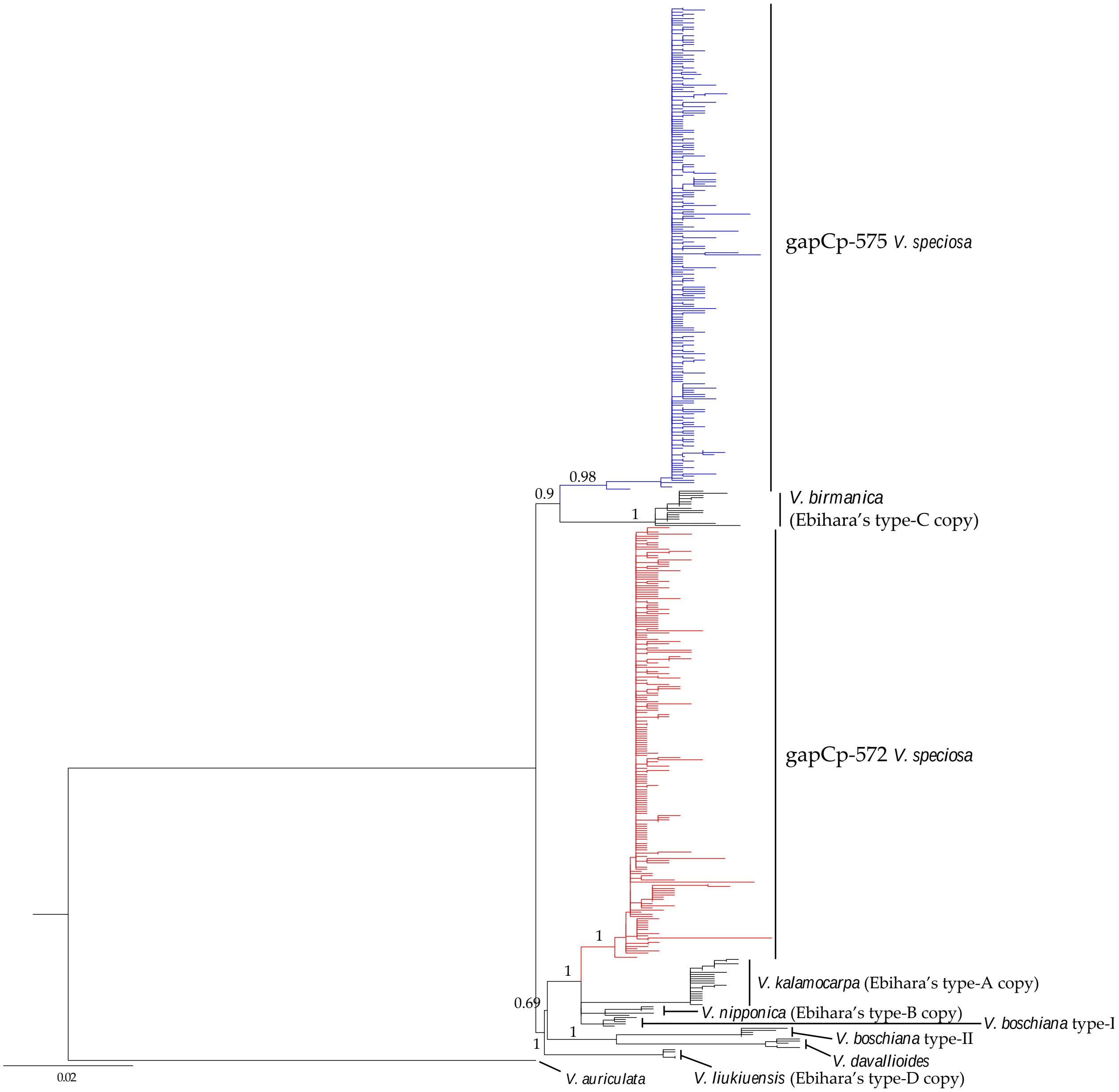

Figure S2. Statistical parsimony networks of the *gapCp* sequences. Networks for the gapCp-572 (upper) and gapCp-575 (lower) copies are shown; the haplotypes are denoted with Roman

numerals; numbers above branches represent the positions of the nucleotide substitution in the aligned sequences; circle sizes are proportional to the number of sequences found for each haplotype.

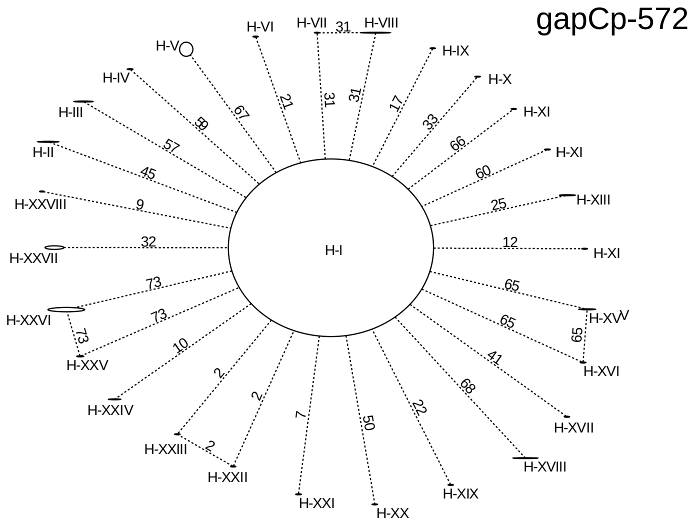

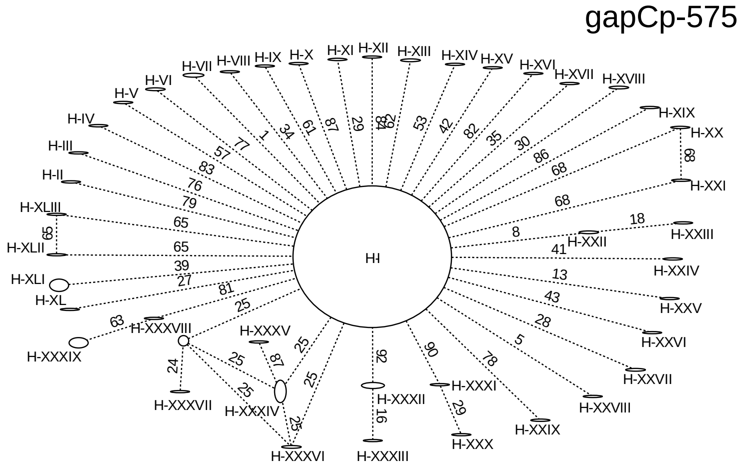

Figure S3. ptDNA haplotype phylogeny derived from the Bayesian inference with MrBAYES. The tree is 50% majority-rule consensus tree of the sample of trees after discarding 25% as burn-in; haplotypes are denoted with Roman numerals; numbers above branches are Bayesian posterior probabilities; for outgroup species, accession numbers of sequences taken from GenBank are shown before the species name.

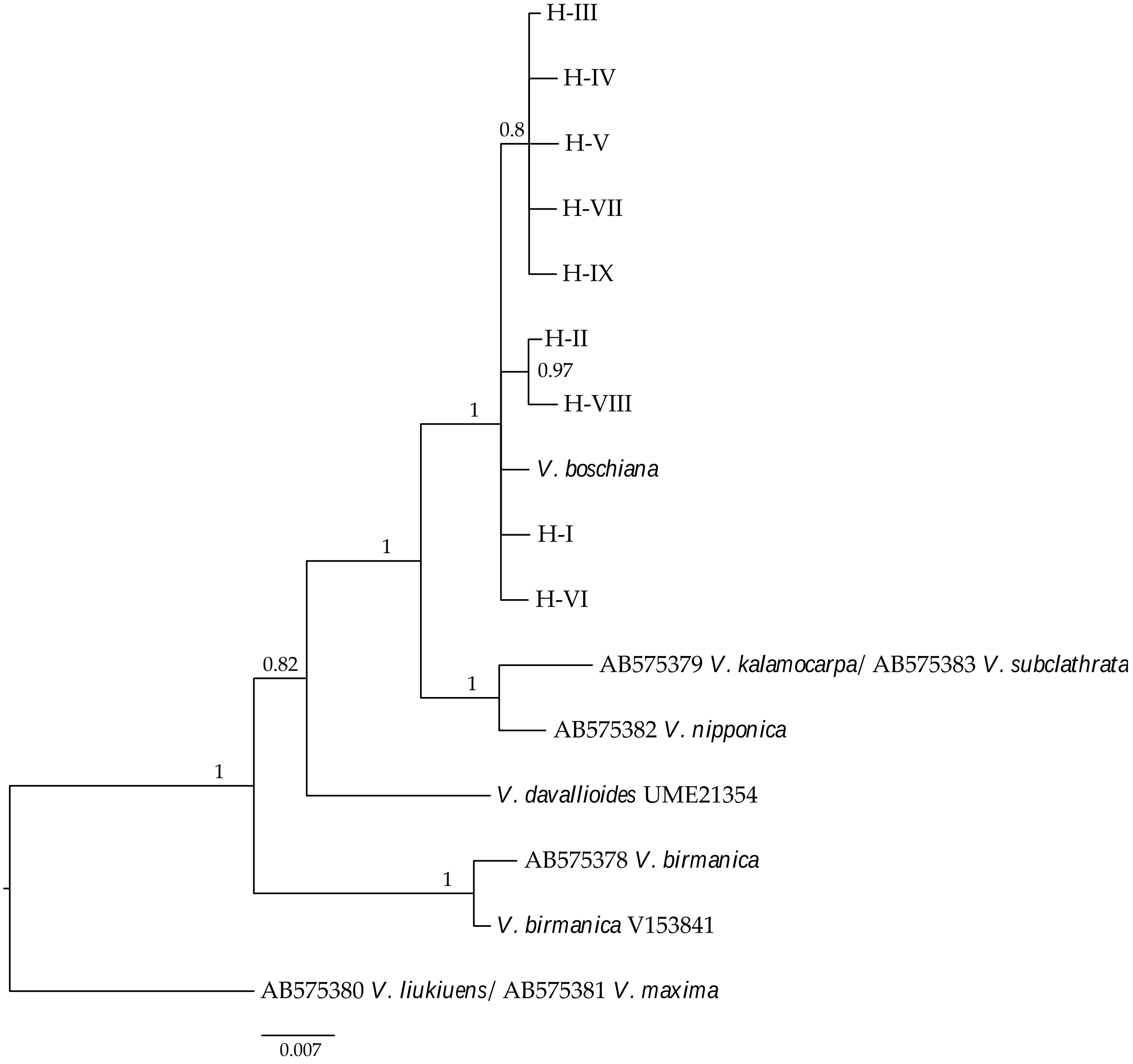

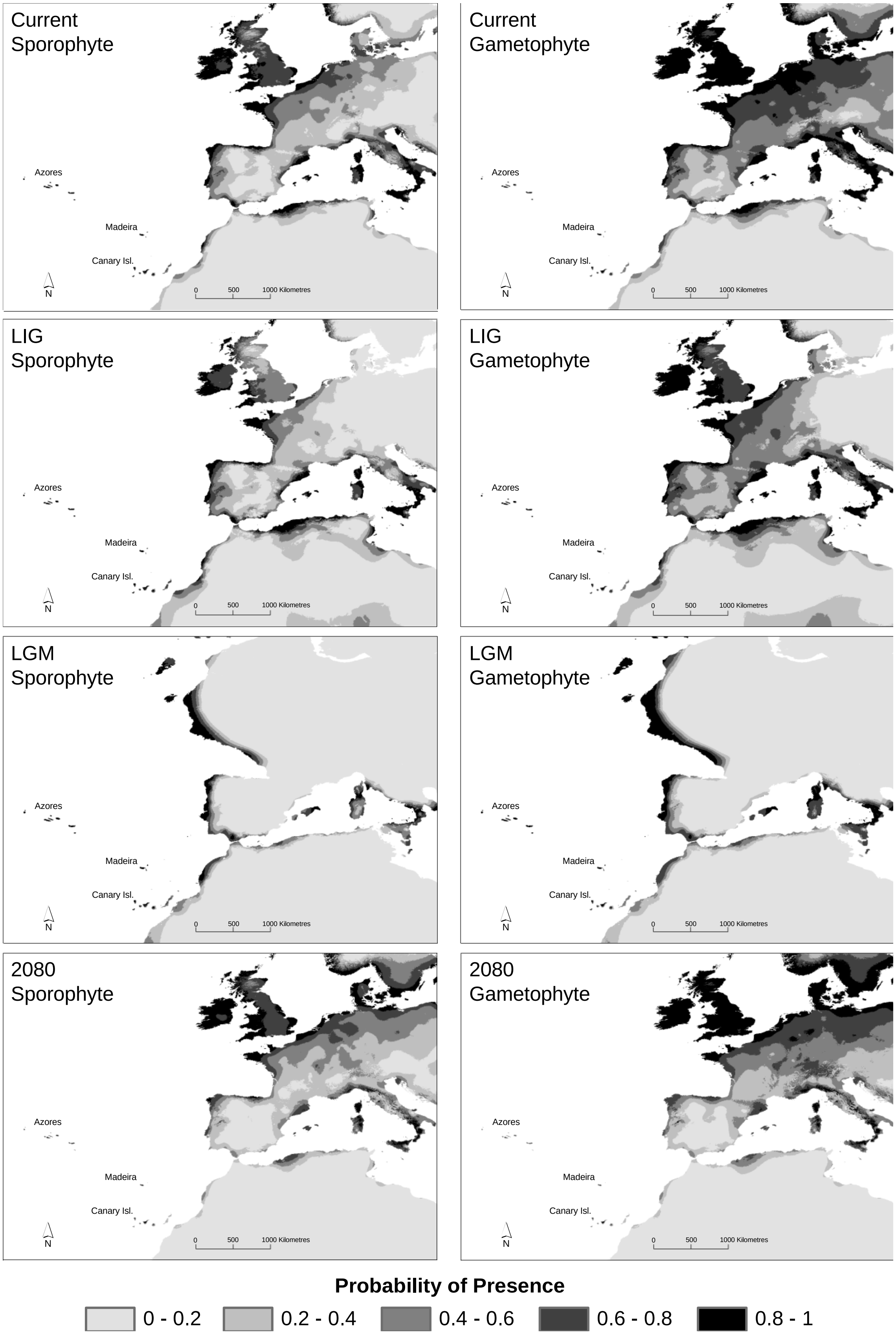

Figure S4. Results of species distribution modelling for the sporophyte and gametophyte phases of the life cycle of *Vandenboschia speciosa* using the maximum entropy algorithm and the Community Climate System Model (CCSM), as implemented in MaxEnt. Projections for current, last interglacial (LIG, *c.* 120-140 ka), last glacial maximum (LGM, *c.* 21 ka), and future (2080) conditions are shown.

Table S1. Sampling details of *Vandenboschia speciosa* populations and outgroup species.

Code	Location	Voucher[a]	Geographical coordinates	Sample size *trn*H-*psb*A	Sample size *gapCp* [b]
Andalusia					
ALM	Almoraima		N36.304°/W5.520°	G: 5	5
COQ	Canuto de Ojén Quesada	*GDA 61589*	N36.127°/W5.585°	G:5/S:11	5
CRM	Cabecera del río de la Miel	*GDA 62522*	N36.105°/W5.528°	S:6	
MCH	Moracha	*GDA 62523*	N36.497°/W5.584°	G:5/S:5	5
SCD	Garganta de la Sauceda	*GDA 62524*	N36.535°/W5.605°	S:3	
SDN	Sierra del Niño	*GDA 62525*	N36.186°/W5.610°	S:4	
VIF	Valdeinfierno	*GDA 62526*	N36.224°/W5.604°	G:5/S:5	5
Azores					
CAR	Terceira: Algar do Carvão		N38.727°/W27.215°	G:5/S:5	5
CID	São Miguel: Sete Cidades		N37.835°/W25.788°	G:5/S:5	5
CON	São Miguel: Lagoa do Congro		N37.754°/W25.407 °	G:5/S:5	5
NAT	Terceira: Gruta do Natal		N38.738°/W27.264°	G:5/S:5	5
Basque Country					
AZK	Azketa erreka		N43.194/W1.940°	G:5/S:5	4
ERR	Erramundi erreka		N43.377°/W1.826°	G:3/S:5	3
ITU	Iturraingo erreka		N43.373°/W1.833°	G:5	2
USO	Usoko erreka		N43.242°/W1.908°	G:5/S:11	5
Canary Isl.					
ANC	La Gomera: Ancón Negro		N28.134°/W17.273°	G:5/S:5	5
CED	La Gomera: Bco. del Cedro		N28.120°/W17.225°	G:4/S:5	4
IJU	Tenerife: Ijuana		N28.560°/W16.172°	G:5/S:5	5
PIJ	Tenerife: El Pijaral		N28.553°/W16.188°	G:5/S:5	5
ZAR	La Gomera: La Zarcita		N28.119°/W17.224°	G:5/S:5	5
Czech Republic					
HAR	Harasov		N50.410°/E14.567°	G:6	3
MUZ	Mužský		N50.528°/E15.054°	G:5	3
SKA	Skalka		N50.585°/E14.424°	G:5	3
Galicia					
EUM	Eume		N43.404°/W8.087°	G:5/S:1	5
SEI	Seixo		N43.706°/W7.946°	G:5/S:5	5
Ire-Wal-Bri*					
COR	Cork		N51.570°/W9.148°	G:5/S:5	5
DEV	Devil’s Bridge		N52.376°/W3.849°	G:4	3
LIM	Limerick		N52.663°/W8.387°	G:5/S:4	5
TAU	Taupont		N47.962°/W2.429°	G:2/S:4	2
WAT	Waterford		N52.115°/W7.585°	G:5/S:5	5
Italy					
SER	Seravezza		N44.015°/E10.219°	G:5/S:6	5
STA	Stazzema		N43.991°/E10.315°	G:5	5
Luxembourg					
ARD	Ardennes		N49.906°/E5.954°	G:5	3
BEA	Beaufort		N49.832°/E6.287°	G:3	3
ROL	Rollingen		N49.739°/E6.133°	G:3	3
Madeira					
FRI	Ribeiro Frio		N32.734°/W16.886°	G:5/S:5	5
POR	Levada Portadela		N32.747°/W16.823°	G:3/S:1	3
URZ	Lombo do Urzal		N32.776°/W16.977°	S:5	
Vosges du Nord					
BIT	Bitche		N49.024°/E7.620°	G:5	3
PIE	La Petite Pierre		N48.848°/E7.301°	G:5	3
V. davallioides[c]		*UME 213054*		S:1	1
		UME 213055		-	4
V. birmanica[c]		*UPS V-153841*		S:1	-
V. boschiana[c]	Personal collection of Fred J. Rumsey	*Sample 1*		S:1	S:5
		Sample 2		S:1	S:5

[a] Voucher available only for sporophytes; [b] *gapCp* only studied with gametophyte individuals except outgroups; [c] species analysed as outgroups. [*]Ireland-Wales-Brittany. G, Gametophyte individuals; S, sporophyte individuals.

Table S2. Percentage contribution and permutation importance of selected model for the species distribution modelling (SDM).

Variable	Gametophyte		Sporophyte		Species as a whole	
	MaxEnt Percent contribution	MaxEnt Permutation importance	MaxEnt Percent contribution	MaxEnt Permutation importance	MaxEnt Percent contribution	MaxEnt Permutation importance
**Min. temperature of coldest month**	51.5	75	46.5	82.1	51.5	74.6
**Mean diurnal range**	40.8	15	41.1	10.9	40.8	13.9
Precipitation of warmest quarter	2.8	0.2	6.4	2.7	2.7	0.3
Precipitation seasonality	2.4	0.6	4.4	2.1	2.3	0.7
max. temperature of warmest month	2.2	5.8	0.6	0	2.3	7.2
Precipitation of coldest quarter	0.2	1.5	0.4	0	0.3	1.3
Precipitation of wettest month	0.1	1.8	0.4	1.8	0.1	2
Precipitation of driest month	0	0	0.1	0.4	0	0

Variables in bold were selected for the final model.

Table S3 Neutrality tests Fu's *F* and Tajima's *D* at the regional and supra-regional groupings.

Code	*trn*H-*psb*A				gapC-572				gapC-575			
	D	*P-value*	*F*	*P-value*	*D*	*P-value*	*F*	*P-value*	*D*	*P-value*	*F*	*P-value*
Andalusia	1.634	0.945	3.412	0.911	-1.732	**0.02**	-3.381	**0.002**	-2.104	**0.005**	-7.578	**<.001**
Azores	-1.411	0.071	-1.592	0.064	-2.103	**0.001**	-6.956	**<.001**	-1.608	0.188	-4.683	N.A.
Basque Country	0.35	0.688	0.462	0.64	-2.006	**0.003**	-5.317	**<.001**	-1.7334	**0.012**	-3.147	**0.002**
Canary Isl.	-0.271	0.421	0.703	0.632	-1.879	**0.008**	-5.036	**0.001**	-1.860	**0.008**	-7.928	**<.001**
Czech Republic	0	1	0	1	-1.491	**0.07**	-1.546	**0.018**	-2.121	**0.002**	-3.415	**0.003**
Galicia	0.735	0.802	1.279	0.789	-1.731	**0.015**	-2.9556	**<.001**	-1.9556	**0.004**	-4.434	**<.001**
Ire-Wal-Bri*	1.503	0.922	1.139	0.760	-1.881	**0.006**	-4.979	**<.001**	-2.349	**<.001**	-10.947	**<.001**
Italy	0	1	0	N.A.	-2.056	**0.003**	-5.655	**<.001**	-1.943	**0.004**	-3.698	**0.001**
Luxembourg	0	1	0	N.A.	-1.706	**0.021**	-2.527	**0.004**	0	1	-0.879	0.078
Madeira	0.541	0.774	2.033	0.797	-1.159	0.155	-0.649	0.105	0	1	0	N.A.
Vosges du Nord	0.019	0.658	1.523	0.727	-1.562	**0.021**	-1.964	**0.008**	-1.697	**0.029**	-2.449	**0.001**
North	-0.224	0.412	-1.018	0.347	-2.366	**<.001**	-340	**<.001**	-2.478	**<.001**	-340	**<.001**
Cantabrian	0.388	0.68	0.667	0.655	-2.239	**<.001**	-11.044	**<.001**	-2.244	**<.001**	-10.426	**<.001**
South	0.447	0.713	1.768	0.802	-2.392	**<.001**	-23.066	**<.001**	-2.399	**<.001**	-28.768	**<.001**

The results for the regional level are shown above the dotted line, and those for the supra-regional level appear below the dotted line. *Ireland-Wales-Brittany. North, Northern evolutionary unit; Cantabrian, Cantabrian Cornice; South, Southern evolutionary unit.

Statistically significant values are indicated in bold text. N.A., not applicable.
